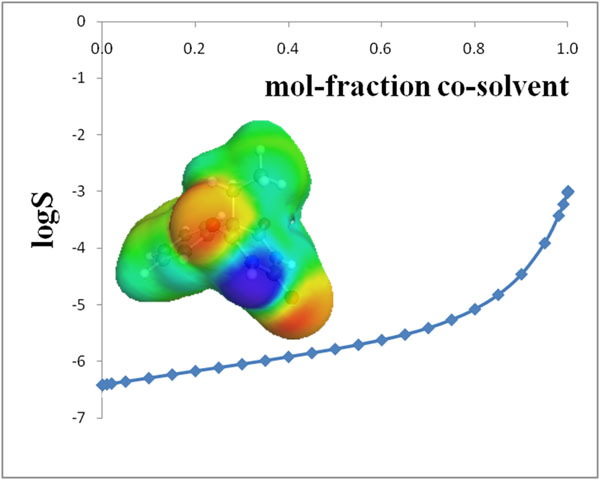# Solvent-screening and co-crystal screening for drug development with COSMO-RS

**DOI:** 10.1186/1758-2946-4-S1-O14

**Published:** 2012-05-01

**Authors:** A Klamt

**Affiliations:** 1 COSMOlogic GmbH&CoKG, Leverkusen and Inst. of Phys. and Theoretical Chemistry, University of Regensburg, Germany

## 

Bringing active pharmaceutical or agrochemical ingredients (APIs) in solution often is the most demanding step in pharmaceutical and agrochemical development. The COSMO-RS method, which has been originally developed by the author during his 12 years at Bayer, is a unique combination of quantum chemical information and liquid phase thermodynamics and currently is proven to be the most accurate method for predicting the free energy of molecules in solution. Based on COSMO-RS theory the COSMOtherm suite of software tools is able to address a broad range of important aspects of solubilization and thus is an ideally suited toolset for rational solubilization development:

- Solvent screening, including mixtures and variable temperatures

- logP, logD and pK_a_ prediction, general multi-phase distribution

- conformational preference and tautomer trends in solution

- Co-crystal screening based on mixing enthalpy

- solubility in micellar systems

- solvent-dependent free energy of crystal faces

**Figure 1:**